# Ammonia Detection Methods in Photocatalytic and Electrocatalytic Experiments: How to Improve the Reliability of NH_3_ Production Rates?

**DOI:** 10.1002/advs.201802109

**Published:** 2019-02-15

**Authors:** Yunxuan Zhao, Run Shi, Xuanang Bian, Chao Zhou, Yufei Zhao, Shuai Zhang, Fan Wu, Geoffrey I. N. Waterhouse, Li‐Zhu Wu, Chen‐Ho Tung, Tierui Zhang

**Affiliations:** ^1^ Key Laboratory of Photochemical Conversion and Optoelectronic Materials Technical Institute of Physics and Chemistry Chinese Academy of Sciences Beijing 100190 P. R. China; ^2^ Center of Materials Science and Optoelectronics Engineering University of Chinese Academy of Sciences Beijing 100190 P. R. China; ^3^ School of Chemical Sciences The University of Auckland Auckland 1142 New Zealand

**Keywords:** ammonia, detection, electrocatalysis, photocatalysis

## Abstract

The enzyme nitrogenase inspires the development of novel photocatalytic and electrocatalytic systems that can drive nitrogen reduction with water under similar low‐temperature and low‐pressure conditions. While photocatalytic and electrocatalytic N_2_ fixation are emerging as hot new areas of fundamental and applied research, serious concerns exist regarding the accuracy of current methods used for ammonia detection and quantification. In most studies, the ammonia yields are low and little consideration is given to the effect of interferants on NH_3_ quantification. As a result, NH_3_ yields reported in many works may be exaggerated and erroneous. Herein, the advantages and limitations of the various methods commonly used for NH_3_ quantification in solution (Nessler's reagent method, indophenol blue method, and ion chromatography method) are systematically explored, placing particular emphasis on the effect of interferants on each quantification method. Based on the data presented, guidelines are suggested for responsible quantification of ammonia in photocatalysis and electrocatalysis.

## 1 Introduction

The Haber–Bosch process for the reduction of nitrogen (N_2_) to ammonia (NH_3_) is one of the fundamental pillars of today's chemical industry.^1^ The process operates under relatively extreme conditions (200–250 bars, 400–500 °C) over an Fe‐based catalyst.[[qv: 1a]] The H_2_ used as the reducing agent in NH_3_ synthesis is typically obtained by steam methane reforming (SMR) coupled with the water–gas shift (WGS) reaction, with the WGS reaction releasing CO_2_ into the atmosphere.^2^ Considering the high energy inputs needed to drive the Haber–Bosch process and SMR, as well as environmental concerns around these processes relating to the their large carbon footprint, more sustainable approaches for N_2_ fixation are demanded. Photocatalysis and electrocatalysis^3^ provide green and sustainable technologies to drive thermodynamically uphill chemical transformations such as water splitting and CO_2_ reduction under ambient conditions, thereby hinting at potential new pathways for NH_3_ synthesis.

Biomolecules containing coordinatively unsaturated metal sites display excellent performance for N_2_ fixation. The enzyme nitrogenase contains both Fe protein and FeMo protein units,^4^ motivating the development of biomimetic analoges such as Mo/Fe sulfides and CdS:MoFe protein systems for N_2_ reduction with water under ambient conditions.^5^ Inspired by these biocatalyst systems, researchers are now exploring the potential of other materials, such as TiO_2_,^6^ BiOBr/Cl,^7^ layered double hydroxides (LDHs),^8^ Bi_4_V_2_O_11_/CeO_2_,^9^ and C‐based materials,^10^ that are also capable of N≡N bond activation via photoinduced charge transfer under light illumination or when applied as electrocatalysts. In these studies, the product (NH_3_) is typically detected by one of a number of possible methods, including spectrophotometric/colorimetric assays (Nessler's reagent and the indophenol blue method),^11^ ion chromatography,^12^ fluorescence,^13^ ammonia ion selective electrodes,^14^ or ^1^H NMR spectroscopy.^15^ For simple solutions of ammonia in water, these methods show high accuracy over a wide range of ammonia concentrations. However, changes in pH or the presence of interferants (impurity ions, other molecules, and especially N‐containing molecules, certain solvents) can adversely impact the accuracy of these methods, especially at the nanomolar/micromolar concentration levels that NH_3_ is typically found in many photocatalytic or electrocatalytic experiments. Indeed, quantification of NH_3_ generated in photocatalytic and electrocatalytic N_2_ fixation studies is especially challenging since various sacrificial agents containing NH*_x_* species (such as triethanolamine) and electrolytes (e.g., KNO_3_) are often introduced into the catalytic system to enhance reaction rates. For photocatalytic nitrogen fixation, the reaction medium typically consists of aqueous solutions containing sacrificial agents (hole scavengers) like methanol, alcohol, and PrOH.^16^ These alcohols and their oxidation products may also form complexes with NH_3_ and impair its quantification (especially if colored complexes are formed leading to an overestimation of the NH_3_ concentration in spectrophotometric assays). Furthermore, the synthesis of many nanomaterial‐based photocatalysts and electrocatalysts involves the use of nitrogen‐containing surface capping agents to achieve a specific morphology. During reaction, some of these capping agents may be lost to the reaction medium, thus complicating the quantification of NH_3_. Further, many photocatalysts are nitrogen‐rich, such as g‐C_3_N_4_, which can add additional complexities when attempting to quantify actual NH_3_ production rates. Accordingly, the use of isotopically labeled ^15^N_2_ to confirm the formation of ^15^NH_3_ via direct N_2_ fixation is highly recommended, with product (^15^NH_4_^+^) detection via ^1^H NMR or mass spectrometry.^17^ It is worth noting that there is low‐level contamination from isotope‐labeled gases due to the special synthetic process of ^15^N_2_, which should be of particular concern.^18^ As pointed out by Medford and Hatzell,[[qv: 6b]] adventitious carbon on the surface of photocatalysts can act as an active participant in nitrogen reduction, promoting reactions that otherwise could not occur on the clean catalyst surface. Many groups further highlight the importance of catalyst cleanliness and the need for careful analysis of catalytic data due to the presence of trace amounts of NH_3_ in air.[[qv: 3c,17,19]] To date, minimal work has been reported on the effects of sacrificial agents, electrolyte, organic ligands, and other contaminants on the reliability and accuracy of NH_3_ quantification. Establishing the range of conditions under which data obtained using each testing method is reliable, especially for NH_3_ concentrations at the ppm (mg L^−1^) and ppb (µg L^−1^) levels, requires systematic studies to be conducted against a broad spectrum of potential interferants. Choice of the best NH_3_ quantification method will likely depend on the photocatalytic or electrocatalytic system under investigation. However, at this stage the rational choice of the best method is not possible and somewhat arbitrary due to the lack of detailed information about the factors, which adversely impact ammonia quantification at nanomolar/micromolar concentrations.

Herein, we aimed to systematically compare three common ammonia detection methods (Nessler's reagent method, the indophenol blue method, and ion chromatography) in terms of their ability to quantify ammonia production following photocatalytic and electrocatalytic N_2_ fixation tests. Our results conclusively demonstrate that the pH of the solution, the presence of certain metal ions, sacrificial agents, and nitrogen‐containing chemicals can all adversely impact ammonia detection and quantification. By comparing the results obtained using the different ammonia detection methods over a range of reaction conditions, we hoped to provide a framework for the selection of the best NH_3_ quantification method for a given set of reaction conditions.

## 2 Results and Discussion

As discussed above, various methods are used for the quantification of ammonia in aqueous media,[[qv: 6a,9,20]] with these same methods also applied to quantify NH_3_ production in photocatalytic and electrocatalytic experiments. Ion chromatography offers many advantages (such as high sensitivity and good reproducibility), though it is expensive and requires complex instrumentation. Accordingly, spectrophotometric methods are more widely used due to their lower cost, of which the Nessler's reagent method and the indophenol blue method are the most common. Nessler's reagent^11^ is a solution containing K_2_HgI_4_ and KOH. Iodide and mercury ions react with ammonia under alkaline conditions to produce a reddish‐brown complex, which absorbs strongly at 420 nm (Equation (1)). The absorbance of the resulting reddish‐brown complex is directly proportional to the ammonia concentration in the absence of interferants. In order to minimize interference from other ions (Fe^3+^, Co^2+^, Ni^2+^, Cr^3^+, Ag^+^, S^2−^, and others), Rochelle salt (KNaC_4_H_4_O_6_ × 4H_2_O) is often added during the detection.

(1) $$2{\left[{\mathrm{HgI}}_{4}\right]}^{2-}+{\mathrm{NH}}_{3}+3{\mathrm{OH}}^{-}\to {\mathrm{Hg}}_{2}{\mathrm{ONH}}_{2}I+7I^{-}+2H_{2}O$$

It is worth noting that: i) mercury ions in Nessler's reagent are toxic and thus the reagent should be used carefully; ii) the lifetime of Nessler's reagent is relatively short (around three weeks); iii) the water used to prepare the Nessler's reagent solution must be free of ammonia (ultrapure water); and iv) the reaction time of ammonia with Nessler's reagent also affects the accurate quantification of NH_3_, with a reaction time from 10 to 30 min being recommended (Figure S1, Supporting Information).

The indophenol blue method follows the Bethelot reaction (Equation (2)),^11^ involving the reaction of ammonia with phenol and hypochlorite in alkaline solution to generate a blue‐colored indophenol product. Sodium nitroprusside is used as a catalyst to intensify the color change in indophenol reaction, with citrate buffer used to stabilize the pH of the reaction solution.


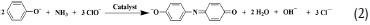


**Figure 1:**
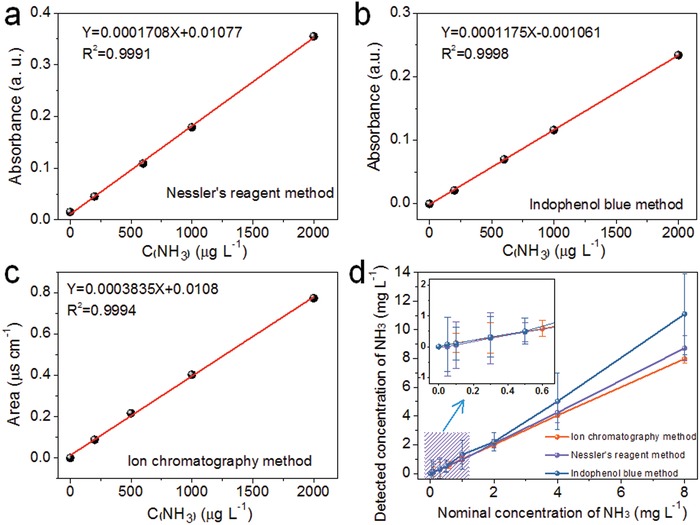
Standard curves for NH_3_ using a) Nessler's reagent, b) indophenol blue, and c) ion chromatography methods. d) Performance comparison of the different methods for ammonia quantification (error bars are based on triplicate measurements).

To explore the detection ranges for NH_3_ using Nessler's reagent, indophenol blue, and ion chromatography, standard curves were prepared and are presented in **Figure** **1**a–c. In the absence of interferants, all three determination methods afforded highly linear responses with NH_3_ concentration in the concentration range 0–2000 µg L^−1^ (*R* ^2^ = 0.9991, 0.9998, and 0.9996, respectively). Figure 1d compares data collected using the three determination methods over a wide NH_3_ concentration range (0–8 mg L^−1^). At low concentrations (0–500 µg L^−1^), all three methods are concordant, with the detected NH_3_ concentration matching closely the nominal ammonia concentration. At concentrations above 500 µg L^−1^, the results from the Nessler's reagent method and the ion chromatography methods are similar and close to the nominal ammonia concentration, whereas the indophenol blue method leads to an overestimation of the ammonia concentration (see the inset of Figure 1d). Results clearly indicate that the Nessler's reagent method and ion chromatography methods are better for aqueous ammonia quantification than the indophenol method over the wide concentration range studied. Simultaneously, the error observed in these ranges (e.g., around 300 µg L^−1^) have a great influence on the calculation of Faraday efficiency in electrocatalysis (Figure S2, Supporting Information). However, all methods are viable at low ammonia concentrations (up to 500 µg L^−1^). Compared with the spectrophotometric methods, the main advantages of ion chromatography method are: 1) high efficiency and convenience (it takes only a short time to simultaneously detect multiple components and cations); 2) high sensitivity (can be used at concentrations ranging from a few µg L^−1^ to several hundred mg L^−1^); 3) high selectivity (inorganic and organic cations can be quantified by selecting appropriate separation and monitoring methods); 4) good stability and high compatibility (the high pH stability of ion chromatography column packings allows the use of strong acids as eluents, which helps expand the range of applications).

**Figure 2:**
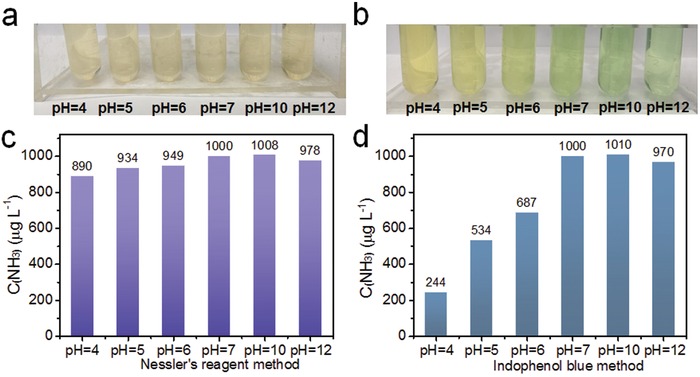
Photographs of ammonia solutions with different pH in the presence of a) Nessler's reagent and b) indophenol blue. c,d) Concentrations of ammonia detected using each method, respectively. The ammonia concentration was 1000 µg L^−1^ in all experiments.

Solution pH is an important factor for the quantification of ammonia. Photocatalytic and electrocatalytic N_2_ fixation reactions are performed in aqueous solutions over a wide range of pH values (typically adjusted with H_2_SO_4_ or NaOH to achieve a desired pH). Thus, the effect of pH of the reaction medium on NH_3_ detection needs serious investigation. Accordingly, comparative tests were performed to investigate the effect of pH on ammonia detection with Nessler's reagent and the indophenol blue method (the details are provided in Supporting Information). As shown in **Figure** **2**a,c, both methods were largely insensitive to pH at pH values of 7 and above, resulting in very small standard errors (<3%). The Nessler's reagent method also worked reasonably well at lower pH, showing an 11% decrease (i.e., 11% relative error) in the amount of ammonia detected at pH 4 compared with pH 7. This slight decrease is attributed to subtle differences in how iodide and mercury ions reacted with ammonia under acidic conditions compared with neutral or alkaline conditions. However, the indophenol blue method displayed an obvious solution change from light yellow at pH 7 to light green at pH 4 (Figure 2b), which severely impacted NH_3_ quantification. Figure 2d revealed that the quantification of NH_3_ in acidic media by the indophenol blue method is highly inaccurate (relative errors are −75.6% at pH 4, −46.6% at pH 5, and −31.3% at pH 6, respectively) due to the instability of NaOCl under acidic conditions. We conclude that the ammonia concentrations can be detected using both methods with good accuracy under neutral or alkaline conditions, but only Nessler's reagent method is suitable for ammonia detection in acidic solution.

**Figure 3:**
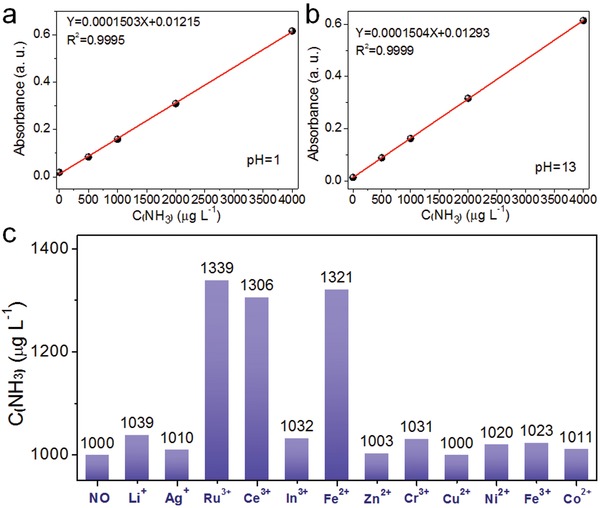
Standard curves for NH_3_ detection with Nessler's reagent at a) pH 1 and b) pH 13. c) The apparent concentration of ammonia detected in the presence of different metal ions. The ammonia concentration was 1000 µg L^−1^ in all experiments. The metal ion concentrations were 0.01 mmol L^−1^.

To further investigate the detection performance of the Nessler's reagent method, standard curves for NH_3_ were prepared at pH 1 and pH 13 (**Figure** **3**a,b). At both pH, strong linear relationships were established between absorbance at 420 nm and the concentration of ammonia in the solution (*R* ^2^ = 0.9995 and 0.9999, respectively). The result confirmed the high detection stability and accuracy of the Nessler's reagent method under both acidic and alkaline conditions. The effect of certain metal ions on the determination of NH_3_ using the Nessler's reagent method in acidic solution was also investigated. A series of 1000 µg L^−1^ ammonia solutions were prepared at pH 1, each containing a different kind of metal ion (0.01 mmol L^−1^). Figure 3c shows the interference effect of different metal ions on the apparent concentration of NH_3_ determined by the Nessler's reagent method. As can be seen in Figure 3c, Ru^3+^, Fe^2+^, In^3+^, and Ni^2+^ exerted an obvious interference effect on ammonia detection, with the corresponding absolute errors (*E*_a_) being +321 µg L^−1^ (Fe^2+^), +300 µg L^−1^ (In^3+^), +135 µg L^−1^ (Ru^3+^), and +124 µg L^−1^ (Ni^2+^). The ions Ag^+^, Ce^3+^, Zn^2+^, Cr^3+^, Cu^2+^, Fe^3+^, and Co^2+^ had negligible effect on ammonia detection. In order to exclude the interference of the color of ions, comparison tests were performed without adding Nessler's reagent and ammonia (results are presented in Table S1 in the Supporting Information). Ru^3+^, In^3+^, and Fe^3+^ ions all showed absorption at 420 nm, whereas the other ions showed negligible absorption at this wavelength. Accordingly, it can be concluded that the interference effect of Ru^3+^, Fe^2+^, In^3+^, and Ni^2+^ arise from absorption by the ions (Ru^3+^, In^3+^, and Fe^3+^) and also chemical reaction between Nessler's reagent and the ions (Fe^2+^, In^3+^, and Ni^2+^) to produce colored compounds. Additionally, the interference effect of higher concentration of metal ions (0.5 m) was examined, with results shown in Figure S3 in the Supporting Information. High concentrations of metal ions nearly resulted in different levels of interference for ammonia quantification. Thus, blank experiments and other quantitative analyses (e.g., ion chromatography or proton NMR spectra) need to be performed in parallel with the Nessler's reagent test to determine the actual concentration of ammonia in acidic media if these particular ions are present.

Conductive carbon is widely used to improve the electrical conductivity of materials in electrocatalysis. However, the conductive carbon itself may be a source of interference for accurate NH_3_ quantification, especially using Nessler's reagent and indophenol blue methods. For comparison (Figure S4, Supporting Information), Carbon (Ketjenblack EC‐300J) was soaked in 0.05 m H_2_SO_4_ for 40 min under ambient conditions. The solution was found to contain almost no ammonia via the Nessler's reagent method, indicating that there is almost no interference from the surface of Carbon. A potentiostatic test was later performed over the same Carbon (Ketjenblack EC‐300J) in Ar saturated 0.05 m H_2_SO_4_ solution over 40 min. The reaction solution was then tested using the Nessler's reagent method. The apparent production rate of ammonia is calculated to be 10.14 µg h^−1^ mg^−1^ with an excellent Faradaic efficiency (50.3%). However, it is not expected that any ammonia formed during the electrocatalytic N_2_ reduction reaction, suggesting that the carbon decomposed into some unknown substance (possible carbon nanoparticles or some compound) during electrochemical process that acted as an interferant. Since Ar was used to degas the reaction solution, N_2_ fixation to NH_3_ during the electrocatalytic reaction can be excluded.^19^

**Figure 4:**
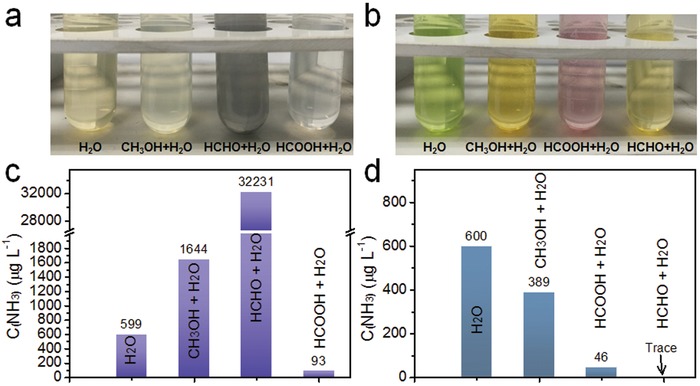
Photographs of different reaction solutions and NH_3_ concentrations determined by a,c) the Nessler's reagent method and b,d) the indophenol blue method in the presence of methanol and its derived oxidation products. The ammonia concentration was 600 µg L^−1^ in all experiments. The sacrificial agent concentration was 40 vol%.

Sacrificial agents, such as methanol, ethanol, and other alcohols, are widely used in the field of photocatalysis as sacrificial hole scavengers to enhance electron–hole pair separation. It was therefore of particular interest to examine the effect of sacrificial agents on ammonia detection using the Nessler's reagent method and indophenol blue method. If methanol is used as a sacrificial agent, it is firstly oxidized to an alpha‐hydroxy radical, then formaldehyde, then formic acid, and finally CO_2_. Accordingly, solutions were prepared by mixing 1000 µg L^−1^ NH_3_ and different solvents (methanol, aqueous formaldehyde, or formic acid) in a 60:40 volume ratio. The final ammonia concentration in each solution was thus 600 µg L^−1^. These solutions were then analyzed using the Nessler's reagent method and the indophenol blue method. **Figure** **4**a,c and Figures S5, S6 in the Supporting Information show data collected for the ammonia solutions detected by the Nessler's reagent method. The color of reaction solutions containing the organic solvents (light green, black, and transparent) were quite distinct from the normal ammonia solution in water (yellow). Clearly, other chemical reactions occurred in the presence of methanol and formaldehyde, with the Hg compounds appearing to be highly soluble in the formic acid solution. The addition of the organic solvents also had a strong interference effect on ammonia detection using the indophenol blue method. The color of reaction solutions change from the standard light green to yellow (with methanol), light pink (with formaldehyde), and light yellow (with formic acid) as shown in Figure 4b,d and Figures S6, S7 in the Supporting Information, respectively. The effect of methanol and the other compounds on NH_3_ quantification using each detection method was pronounced (Figure 4b,d), thereby demonstrating that sacrificial agents can lead to wildly inaccurate estimations of the NH_3_ concentration (in the case of Nessler's reagent method, the NH_3_ concentration was overestimated by ≈53 times in the presence of formaldehyde). Since methanol is a commonly used sacrificial agent in photocatalysis, further experiments were conducted to investigate the interference effect of methanol concentration on the quantification of ammonia in solution. As shown in **Table** **1**, the relative error (*E*_r_) for NH_3_ detection increased to 174% when the concentration of methanol was increased from 0 to 40 vol% (Figure S8, Supporting Information), suggesting that any methanol present during photocatalytic N_2_ fixation experiments would likely interfere with the quantification of NH_3_, resulting in an overestimation of the NH_3_ concentration.Similarly, other sacrificial agents such as ethanol, acetone, isopropanol, formamide, *N*,*N*‐dimethylformamide (DMF), dimethyl sulfoxide (DMSO), or triethanolamine were also found to act as interferants (Figures S9 and S10, Supporting Information). Based on these results, the Nessler's reagent method and the indophenol blue method are not ideal for NH_3_ quantification when sacrificial agents (organic reagents) are used in photocatalytic or N_2_ fixation experiments. If sacrificial agents are to be used, then detailed control experiments must also beperformed to allow the effect of the sacrificial agents on the NH_3_ detection to be fully understood. Certain sacrificial agents can be damaging for the cation chromatography column used in ion chromatography method, further highlighting the need for care when attempting to detect ammonia in the aqueous reaction media of photocatalytic tests.

**Table 1:** The interference effect of different sacrificial agents on ammonia detection by the Nessler's reagent method

Sample	C(NH_3_) [µg L^−1^]a)	Relative error (*E*_r_) [%]
NOb)	598	−0.33
1% methanol	656	+9.33
10% methanol	787	+31.17
20% methanol	1.47 × 10^3^	+145.83
40% methanol	1.64 × 10^3^	+174.00
40% formaldehyde	3.22 × 10^4^	+5.27 × 10^3^
40% formic acid	93	−84.50
40% ethanol	2.66 × 10^4^	+4.34 × 10^3^
40% acetone	80	−86.67
40% isopropanol	7.30 × 10^4^	+1.21 × 10^4^
40% formamide	181	−69.83
40% DMF	1.59 × 10^3^	+165.50
40% DMSO	3.56 × 10^3^	+493.33
40% triethanolamine	9.62 × 10^3^	1.50 × 10^3^

^a)^ 600 µg L^−1^ ammonia standard solution

^b)^ Blank experiment without any other sacrificial agents.

**Figure 5:**
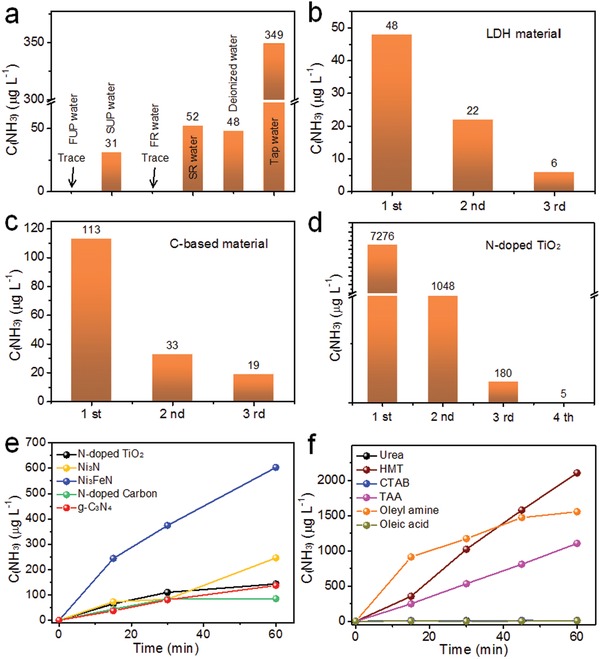
The ammonia concentration determined by ion chromatography for a) different types water (FUP is fresh ultrapure water, SUP is stale ultrapure water, FR is fresh redistilled water, and SR is stale redistilled water), and b–d) aqueous solutions containing LDH, C‐based materials, and N‐doped materials before and after washing with ultrapure water. e,f) Time course of NH_3_ evolution in an Ar atmosphere under UV–vis light irradiation (200–800 nm) for various substances used in catalyst synthesis, as well as a g‐C_3_N_4_ photocatalyst and N‐containing materials.

Since water is the main medium in which ammonia detection is required, additional experiments were undertaken using different types of water (tap water, redistilled water, deionized water, and ultrapure water) via ion chromatography. **Figure** **5**a shows that the ammonia concentration of tap water (349 µg L^−1^) is much higher than that of stale ultrapure water (31 µg L^−1^), stale redistilled water (52 µg L^−1^), and deionized water (48 µg L^−1^). No ammonia was detected in fresh ultrapure water or fresh redistilled water, and thus fresh ultrapure water or fresh redistilled water should be used in photocatalytic and electrocatalytic nitrogen fixation experiments.

The presence of chemisorbed or physisorbed ammonia or amino groups on photocatalysts or electrocatalysts before reaction can also interfere with ammonia detection. Accordingly, it is recommended that the catalysts be washed repeatedly with ultrapure water before testing until no ammonia signal can be detected in the washings. Herein, a range of different photocatalytic and catalytic materials (such as LDH, C‐based materials, N‐doped materials, and metal nitride) were used to investigate to study the effect of washing treatments on NH_3_ removal (Figure 5b–d; Figures S11–S13, Supporting Information). Itwas established that all these catalysts absorbed a small amount of ammonia on their surface, especially the C‐based materials and N‐doped materials, though the adsorbed ammonia could easily be removed over several washing cycles. Some common substances used in the synthesis of photocatalysts as capping agents were also studied. Control experiments using 1 mmol of N‐containing materials in water were conducted in an argon (Ar) atmosphere under UV–vis light irradiation (200–800 nm) (Figure 5e). Hexadecyl trimethyl ammonium bromide (CTAB), urea, and oleic acid showed negligible ammonia production (Figure 5f). On the contrary, oleyl amine, thioacetamide (TAA), and hexamethylenetetramine (HMT) rapidly decomposed into ammonia and other products under light irradiation, indicating that these substances containing NH*_x_* species were unstable during standard photocatalytic testing conditions. In addition, control experiments with g‐C_3_N_4_ and other N‐contained materials in an Ar atmosphere were also conducted, with the concentration of ammonia increasing with time under UV–vis irradiation. The decomposition of g‐C_3_N_4_ 21 and chemisorbed ammonia are therefore also a potential source of NH_3_ during photocatalytic or electrocatalytic studies. Therefore, to have confidence in NH_3_ production data during photocatalytic and electrocatalytic tests, it is recommended that reagents or catalytic materials containing NH*_x_* functional groups are not utilized. To prove that the supplied N_2_ was converted into NH_3_ rather than the NH_3_ evolving from other sources, additional control experiments should be carried out in the presence of Ar and ^15^N_2_.

**Figure 6:**
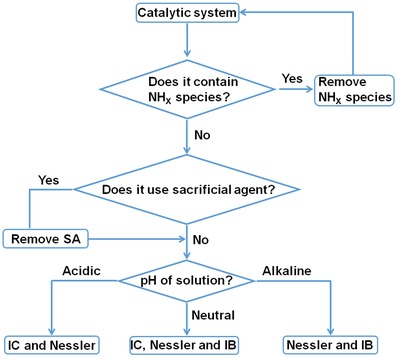
Flowchart showing the criteria that need to be considered when selecting a suitable method for ammonia detection (IC = ion chromatography method, Nessler = Nessler's reagent method, and IB = indophenol blue method).

Based on the data presented above, some useful recommendations can be made regarding the accurate determination of ammonia in the electrocatalytic and photocatalytic tests. These recommendations are summarized in **Figure** **6**: 1) if the photocatalyst or electrocatalyst is to be used for nitrogen fixation, the material itself should not contain any NH*_x_* species before reaction. If the material does contain some NH*_x_* species on the surface, the material should be washed repeatedly with ultrapure water along with other treatments (e.g., ultrasonic cleaning) to minimize NH*_x_* groups which might give a misleading NH_3_ determination. It is recommended that blank and control experiments should be performed to identify all possible sources of NH_3_ in the photocatalytic or electrocatalytic experiments. 2) There are trace amounts of NH_3_ in air and most types of water exposed to air, thus fresh deionized or fresh ultrapure water should be used in N_2_ fixation experiments. 3) Choice of ammonia determination method should be based on the concentration of ammonia produced during the photocatalytic or electrocatalytic reaction (nanomolar/millimolar). Results here demonstrate that the Nessler's reagent method, indophenol blue method, and ion chromatography method are all accurate at NH_3_ concentrations less than 500 µg L^−1^, but the indophenol blue method is less accurate at higher concentrations as well as in acidic media. Further, it is recommended that more than one detection method is used for NH_3_ quantification, with the Nessler's reagent method and ion chromatography being the methods of choice in acidic media and Nessler's reagent method and indophenol blue being preferred in alkaline media. 4) In photocatalysis, sacrificial agents are commonly used as hole scavengers. For photocatalytic N_2_ fixation, the use of sacrificial agents is not recommended since they can interfere with the accurate determination of NH_3_ using spectrophotometric detection methods. 5) In electrocatalysis, the pH of reaction medium can vary dramatically, requiring appropriate selection of NH_3_ detection method. Further, the electrolyte and the presence of certain metal ions can act as interferants, demanding blank experiments be performed with no N_2_ present to allow the effects of the interferants to be gauged. 6) As there is trace amount of NH_3_ in air, experiments with isotopically labeled ^15^N_2_ are crucial to verify the origin of nitrogen in the final product (Figure S14, Supporting Information).

## 3 Conclusion

In summary, we have explored the advantages and limitations of various common detection methods for ammonia quantification (Nessler's reagent method, indophenol blue method, and ion chromatography method) in the context of photocatalytic and electrocatalytic N_2_ fixation. A range of interferants were identified, and strategies for minimizing their impact on NH_3_ quantification were suggested. Data presented were used to develop a flowchart that can be used by researchers to select appropriate NH_3_ detection methods for a particular photocatalytic or electrocatalytic experiment. It is anticipated that this work will provide a useful resource for researchers moving into the field of N_2_ fixation, allowing them to avoid many of the common pitfalls associated with NH_3_ detection and quantification. Further, we hope that this work encourages the development of improved and faster characterization techniques for ammonia detection.

Determination of NH_3_ by the indophenol blue method: Ten milliliters of the ammonia contining‐solution was added to 500 µL of C_4_H_4_(OH)COOH (50 g L^−1^). Then, 100 µL of NaClO (4.5%) and 100 µL of Na_2_Fe(CN)_5_NO·2H_2_O (10 g L^−1^) were successively added into the above solution. Absorbance measurements were performed after 1 h in a 10 mm quartz cuvette at a wavelength of 697.5 nm.

Determination of NH_3_ by the Nessler's reagent method: Ten millilitres of the ammonia‐containing solution was added to an aqueous solution of potassium tartrate (KNaC_4_H_6_O_6_, 0.5 mL, 500 g L^−1^). Then, Nessler's reagent (0.5 mL) was added to the above solution, and the soultion mixed thoroughly. After 10 min, 3 mL of the solution was pipetted into a 10 mm quartz cuvette. Absorbance measurement was performed at λ = 420 nm.

Determination of NH_3_ (^1^H NMR): One millimolar maleic acid was used as the internal standard; 20% DMSO‐d_6_ was used as the solvent. NMR measurements were done on an Agilent 400‐MHz system.

## Conflict of Interest

The authors declare no conflict of interest.

## Supporting information

Supplementary

## References

[1] a) S. Licht , B. Cui , B. Wang , F.‐F. Li , J. Lau , S. Liu , Science 2014, 345, 637; 25104378 10.1126/science.1254234

[2] C. J. M. van der Ham , M. T. M. Koper , D. G. H. Hetterscheid , Chem. Soc. Rev. 2014, 43, 5183. 24802308 10.1039/c4cs00085d

[3] a) C. Guo , J. Ran , A. Vasileff , S.‐Z. Qiao , Energy Environ. Sci. 2018, 11, 45;

[4] V. K. Shah , W. J. Brill , Proc. Natl. Acad. Sci. U. S. A. 1977, 74, 3249. 410019 10.1073/pnas.74.8.3249 PMC431518

[5] K. A. Brown , D. F. Harris , M. B. Wilker , A. Rasmussen , N. Khadka , H. Hamby , S. Keable , G. Dukovic , J. W. Peters , L. C. Seefeldt , Science 2016, 352, 448. 27102481 10.1126/science.aaf2091

[6] a) H. Hirakawa , M. Hashimoto , Y. Shiraishi , T. Hirai , J. Am. Chem. Soc. 2017, 139, 10929; 28712297 10.1021/jacs.7b06634

[7] a) H. Li , J. Shang , Z. H. Ai , L. Z. Zhang , J. Am. Chem. Soc. 2015, 137, 6393; 25874655 10.1021/jacs.5b03105

[8] Y. Zhao , Y. Zhao , G. I. Waterhouse , L. Zheng , X. Cao , F. Teng , L. Z. Wu , C. H. Tung , D. O'Hare , T. Zhang , Adv. Mater. 2017, 29, 1703828. 10.1002/adma.201703828 28960530

[9] C. Lv , C. Yan , G. Chen , Y. Ding , J. Sun , Y. Zhou , G. Yu , Angew. Chem. 2018, 130, 6181. 10.1002/anie.201801538 29473991

[10] a) S. Chen , S. Perathoner , C. Ampelli , C. Mebrahtu , D. Su , G. Centi , Angew. Chem. 2017, 129, 2743; 10.1002/anie.201609533 28128489

[11] L. Zhou , C. E. Boyd , Aquaculture 2016, 450, 187.

[12] D. H. Thomas , M. Rey , P. E. Jackson , J. Chromatogr. A 2002, 956, 181. 12108649 10.1016/s0021-9673(02)00141-3

[13] Y. Zhu , D. Yuan , H. Lin , T. Zhou , Anal. Lett. 2016, 49, 665.

[14] A. LeDuy , R. Samson , Biotechnol. Lett. 1982, 4, 303. 10.1002/bit.260240822 18548452

[15] J. Liu , M. S. Kelley , W. Wu , A. Banerjee , A. P. Douvalis , J. Wu , Y. Zhang , G. C. Schatz , M. G. Kanatzidis , Proc. Natl. Acad. Sci. U. S. A. 2016, 113, 5530. 27140630 10.1073/pnas.1605512113 PMC4878479

[16] X. Gao , Y. Wen , D. Qu , L. An , S. Luan , W. Jiang , X. Zong , X. Liu , Z. Sun , ACS Sustainable Chem. Eng. 2018, 6, 5342.

[17] a) L. F. Greenlee , J. N. Renner , S. L. Foster , ACS Catal. 2018, 8, 7820;

[18] R. Dabundo , M. F. Lehmann , L. Treibergs , C. R. Tobias , M. A. Altabet , P. H. Moisander , J. Granger , PLoS One 2014, 9, e110335. 25329300 10.1371/journal.pone.0110335 PMC4201487

[19] G. T. Richardson , J. A. Davies , J. G. Edwards , Fresenius' J. Anal. Chem. 1991, 340, 392.

[20] H. Li , J. Shang , Z. Ai , L. Zhang , J. Am. Chem. Soc. 2015, 137, 6393. 25874655 10.1021/jacs.5b03105

[21] H. Yu , R. Shi , Y. Zhao , T. Bian , Y. Zhao , C. Zhou , G. I. N. Waterhouse , L. Z. Wu , C. H. Tung , T. Zhang , Adv. Mater. 2017, 29, 1605148. 10.1002/adma.201605148 28185339

