# Supplementary material for: Ammonia Detection Methods in Photocatalytic and Electrocatalytic Experiments: How to Improve the Reliability of NH3 Production Rates?
Source: Adv Sci (Weinh). 2019 Feb 15;6(8):1802109. doi: 10.1002/advs.201802109 (PMC6468971; doi:10.1002/advs.201802109)
Supplement: Supplementary file 1 — Supplementary [file ADVS-6-1802109-s001.pdf]

Copyright WILEY-VCH Verlag GmbH & Co. KGaA, 69469 Weinheim, Germany, 2019.

ADVANCED SCIENCE
Open Access

Supporting Information

for *Adv. Sci.,* DOI: 10.1002/advs.201802109

Ammonia Detection Methods in Photocatalytic and Electrocatalytic Experiments: How to Improve the Reliability of NH_3 Production Rates?

*Yunxuan Zhao, Run Shi, Xuanang Bian, Chao Zhou, Yufei Zhao, Shuai Zhang, Fan Wu, Geoffrey I. N. Waterhouse, Li-Zhu Wu, Chen-Ho Tung, and Tierui Zhang**

Copyright WILEY-VCH Verlag GmbH & Co. KGaA, 69469 Weinheim, Germany,

2019.

Supporting Information

Ammonia Detection Methods in Photocatalytic and Electrocatalytic Experiments: How to Improve the Reliability of NH_3 Production Rates?

*Yunxuan Zhao, Run Shi, Xuanang Bian, Chao Zhou, Yufei Zhao, Shuai Zhang, Fan Wu, Geoffrey I.N. Waterhouse, Li-Zhu Wu, Chen-Ho Tung and Tierui Zhang**

Materials:

$AgNO_3$, LiCl, $RuCl_3·3H_2O$, $Ce(NO_3)_3·6H_2O$, $In(NO_3)_3·6H_2O$, $Fe(NO_3)_3·9H_2O$, $FeCl_2·4H_2O$, $Co(NO_3)_2·6H_2O$, $Zn(NO_3)_2·6H_2O$, $Cr(NO_3)_3·9H_2O$, $Cu(NO_3)_2·3H_2O$, $Ni(NO_3)_2·6H_2O$, methanol, ethanol, acetone, isopropanol, formamide, dimethyl formamide (DMF), dimethylsulfoxide (DMSO), triethanolamine, hexadecyltrimethylammonium bromide (CTAB), urea, oleic acid, oleylamine, thioacetamide (TAA), hexamethylenetetramine (HMT), NaOH and HCl (37 wt.%) were obtained from Aladdin and Beijing Chemical Works. $(^{15}NH_4)_2SO_4$ and $^{14}NH_4Cl$ were obtained from Aladdin. All reagents were analytical grade and used without further purification. Argon gas (99.999 %) was obtained from Beijing Qianxi Gases Company (Beijing, China). Ultrapure distilled water was used in all experiments. Ketjenblack ® EC-300J was obtained from Shanghai Buding Chemical Co., Ltd. A tap water sample was obtained from a domestic residence in Beijing.

Synthesis of LDH materials:

The LDHs nanosheets were prepared using a co-precipitation method.[1] The method used a modified colloid mill to achieve rapid mixing and nucleation processes, followed by a separate hydrothermal aging step at 120 ^{o}C for 12 h.

Synthesis of C-based materials:

C-based materials were synthesized using a microwave method.[2] Typically, 100 mg of tungstic acid, 2 g of glucose and 10 mL of PEG-200 were dissolved into 3 mL of distilled water to form a mixed solution. The mixed solution was then heated in 300 W microwave for 2.5 min. The precipitates formed were filtered, washed with water and ethanol, and finally dried at 60 ^{o}C for 24 h.

Synthesis of N-doped TiO_2:

Commercial TiO_2 (P25) (300 mg) was placed in an alumina tube furnace and then heated to 500 °C in an atmosphere of flowing NH_3 (30 mL min^{-1}) at a heating rate of 5 ^{o}C min^{-1}. After ammonolysis at 500 ^{o}C for 3 h, the sample was cooled to room temperature, and the product was collected.

Synthesis of N-doped Carbon:

Zn, Co-ZIF[3] (400 mg) was placed in an alumina tube furnace and then heated to 900 ^{o}C in an atmosphere of flowing NH_3 (30 mL min^{-1}) at a heating rate of 5 °C min^{-1}. After ammonolysis at 900 ^{o}C for 2 h, the sample was cooled to room temperature and the product was collected.

Synthesis of Ni_3N and Ni_3FeN:

Commercial NiO (300 mg) or NiFe-LDH (300 mg) was placed in an alumina tube furnace and then heated to 500 ^{o}C in an atmosphere of flowing NH_3 (30 mL min^{-1}) at a heating rate of 5 ^{o}C min^{-1}. After ammonolysis at 500 ^{o}C for 4 h, samples were cooled to room temperature and the product was collected.

Synthesis of g-C_3N_4:

g-C_3N_4 was obtained using a standard thermal polymerization method.[4]

Preparation of ammonia solutions with different pH:

Ammonia solutions with different pH were prepared by adding an appropriate amount of 28 wt.%. NH_3 to a specific volume of water, followed by pH adjustment with either aqueous H_2SO_4 (0.05 mol L^{-1}) or aqueous NaOH (1 mol L^{-1}). The final concentration of ammonia in all solutions was 1000 μg L^{-1}.

NH_3 production and control experiments:

A series of experiments were performed under UV-visible light irradiation (200-800 nm) supplied by a 300 W Xe lamp (CEL-HXF 300). Typically, photocatalyst or potential interferant (1 mmol) were dispersed in 100 mL of ultrapure water in a 150 mL quartz reactor. The reactor was equipped with a circulating water outer jacket in order to maintain at a constant temperature of 25 ^{o}C. The photocatalyst suspension or solutions containing the

possible interferants were stirred continuously in the dark whilst either high-purity N_2 or high purity Ar was bubbled through the suspension at a flow rate of 60 mL min^{-1} for 10 min. The reactor was then irradiated using the 300 W Xe lamp under either a N_2 or Ar flow (100 mL min^{-1}). At regular intervals, 3 mL aliquots of the reaction solution were collected using a syringe, then immediately centrifuged to remove any solid material. The concentrations of NH_3 was determined by ion chromatography (930 compact IC Flex, Metrohm). Errors in the data were expressed as follows:

The relative error: $Er = \frac{X-M}{M} \times 100\%$, where X is the measured value and M is the true value.

The absolute error: $Ea = X - M$, where X is the measured value and M is the true value.

The faradaic efficiency was calculated according to the following equation:[5]

The faradaic efficiency = $3 \times F \times [NH_3] \times V/(17 \times Q) \times 100\%$

where $[NH_3]$ is the measured NH_3 concentration; V is the volume of the cathodic reaction solution for NH_3 collection; t is the potential applied time; A is the geometric area; m is the loaded mass of catalyst; F is the Faraday constant; and Q is the quantity of applied electricity.

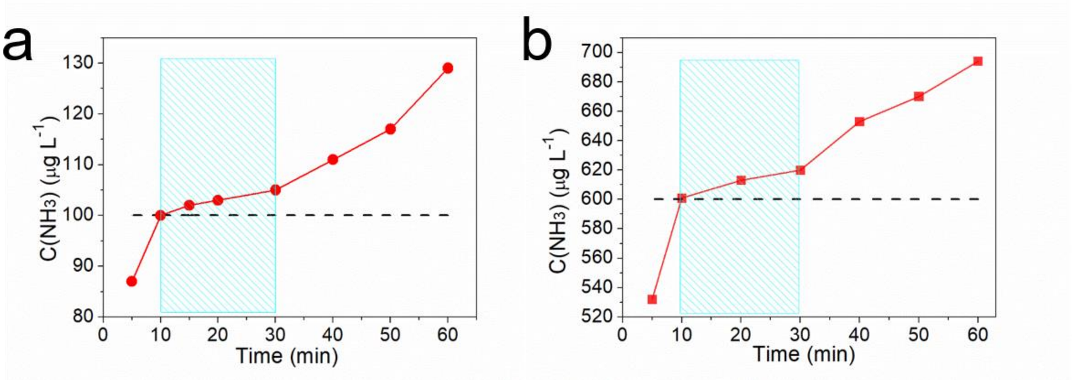

Figure S1. Time course of detected NH_3 by Nessler's reagents method for a certain concentration of aqueous ammonium solution (a): 100 μg L^{-1} and (b): 600 μg L^{-1}.

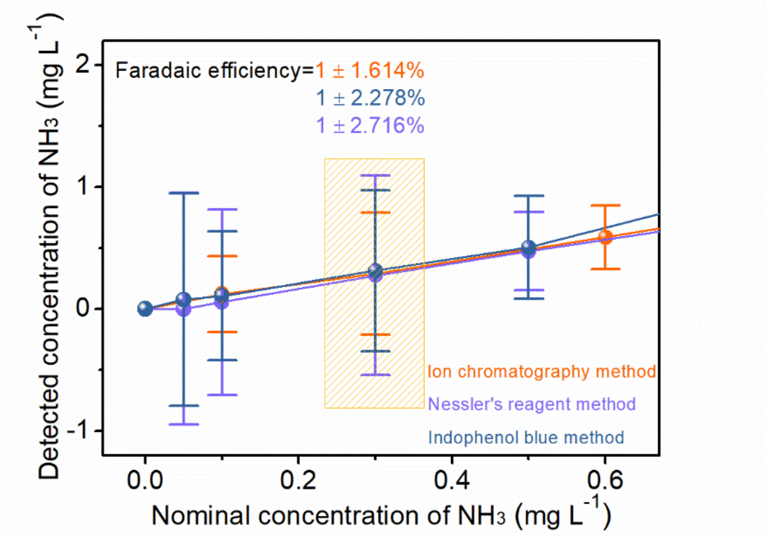

Figure S2. The Faradaic efficiency based on different methods of ammonia quantification.

For a Faradaic efficiency of 1%, the NH_3 yield is estimated to be about 300 μg L^{-1}. Figure S2 shows that the different methods of ammonia quantification afford different Faradaic efficiencies: 1.614% for ion chromatography method, 2.278% for the indophenol blue method and 2.716% for Nessler's reagent method. Thus, longer reaction time are recommended, thereby allowing more accurate ammonia quantification.

Table S1 The effect of different metal ions on ammonia detection by the UV method without Nessler's reagent under acidic conditions.

Sample	Absorbance	Concentration $^{\alpha}$ (μg L^{-1})
No metal ions $^{\beta}$	0-0.001	0
$Ag^{+\ \gamma}$	0.001	0
Ru^{3+}	0.020	126
Ce^{3+}	0.001	0
In^{3+}	0.005	27
Fe^{2+}	0.001	0
Zn^{2+}	0.001	0
Cr^{3+}	0.001	0
Cu^{2+}	0.001	0
Ni^{2+}	0.001	0
Fe^{3+}	0.003	13
Co^{2+}	0.001	0

α: Y=0.0001504X+0.01293, where Y is the apparent NH_3 concentration in mg L^{-1} and X is the absorbance value.
β: Acid solution (pH 1) without any metal ions and Nessler's reagent.
γ: Acidic solution (pH 1) containing different metal ions (0.01 mmol L^{-1}) without adding Nessler's reagent and ammonia.

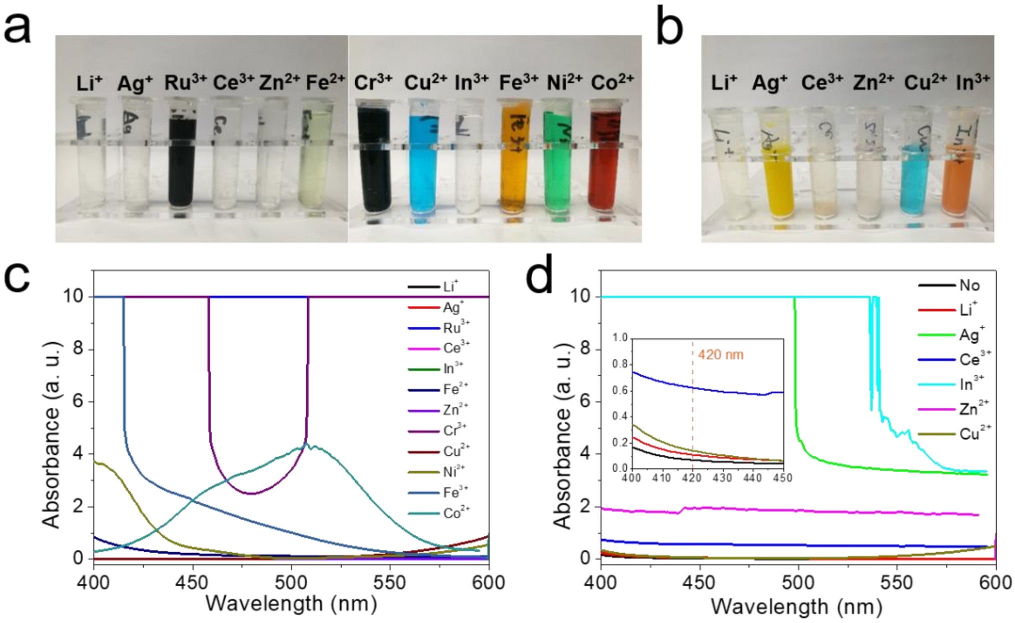

Figure S3. Photographs of ammonia solutions containing high metal ion concentrations (0.5 M) before (a) and after (b) adding Nessler's reagent. Corresponding UV-vis absorption spectra before (c) and after (d) adding Nessler's reagent.

The UV-vis absorption spectra of aqueous solution containing certain metal ions (i.e. 0.5 M solutions of Ru^{3+}, $Fe^{2/3+}$, Cr^{3+}, Ni^{2+} and Co^{2+}) without Nessler's reagent show obvious absorption at 420 nm. Hence, these metal ions at high concentration could interfere with the accurate quantification of NH_3. Further, adding Nessler's reagent to solutions containing other metal ions in high concentration (i.e. 0.5 M solutions of Li^{+}, Ag^{+}, Ce^{3+}, In^{3+}, Zn^{2+} and Cu^{2+}) caused some absorption at 420 nm, especially solutions contained Ag^{+}, Ce^{3+}, In^{3+} or Zn^{2+} for which a precipitate formed, leading to cloudiness and therefore interference detection. Although solutions of Li^{+} and Cu^{2+} ions did not form a precipitate, these metal ions still interfere with ammonia detection.

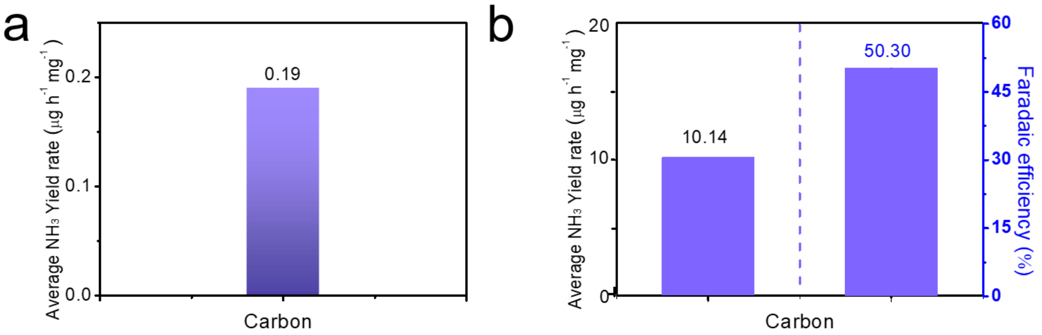

Figure S4. (a) Ammonia evolution rate for carbon materials (Ketjenblack®EC-300J) soaked in 0.05 M H_2SO_4 for 40 min under ambient conditions. (b) Ammonia evolution rate and Faradaic efficiency for carbon materials at -0.2 V versus RHE in Ar atmosphere for 40 min.

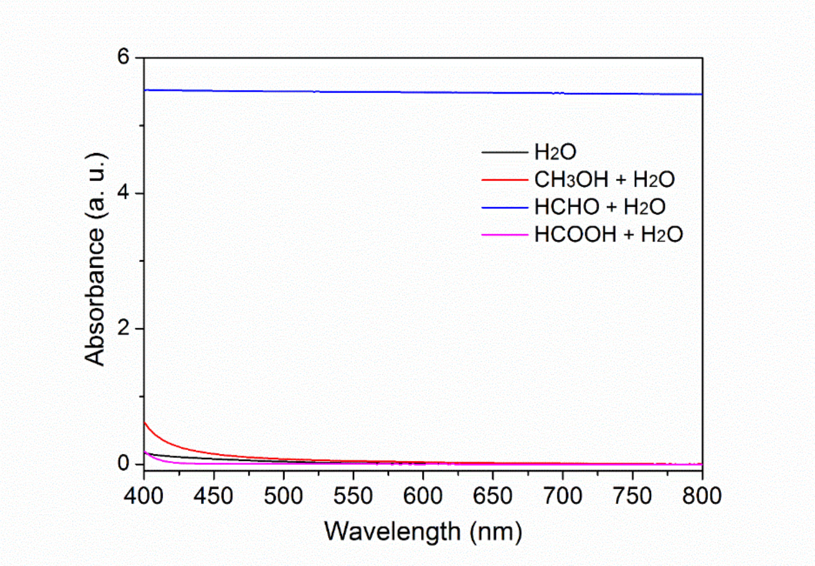

Figure S5. UV-vis absorbance spectra for ammonia solutions at the same concentration (600 μg L^{-1}) in the presence of different organic solvents detected by Nessler's reagent method.

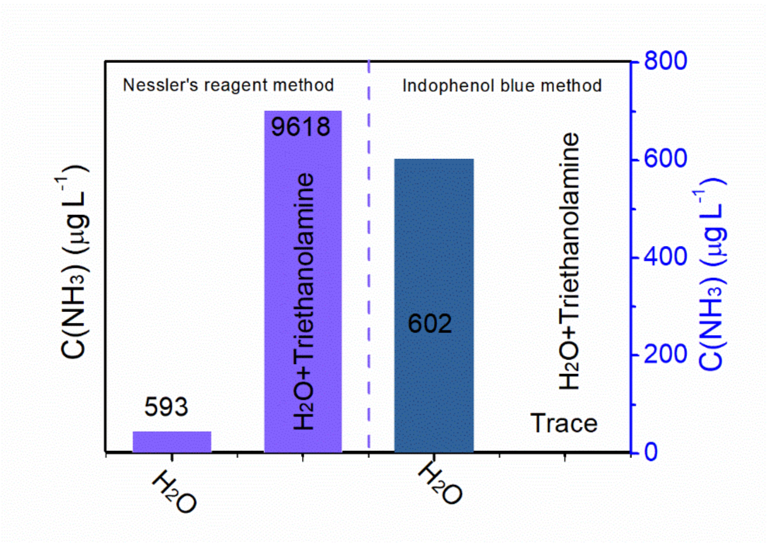

Figure S6. The apparent concentration of ammonia detected in the presence of triethanolamine. The ammonia concentration was 600 μg L^{-1} in all experiments. The sacrificial agent concentration was 40 vol.%.

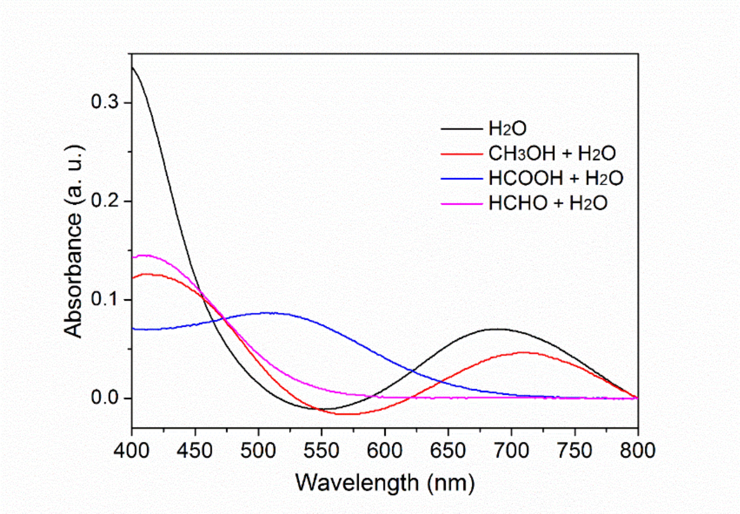

Figure S7. UV-vis absorbance spectra for ammonia solutions of the same concentration (600 μg L^{-1}) containing different organic solvents detected by the indophenol blue method.

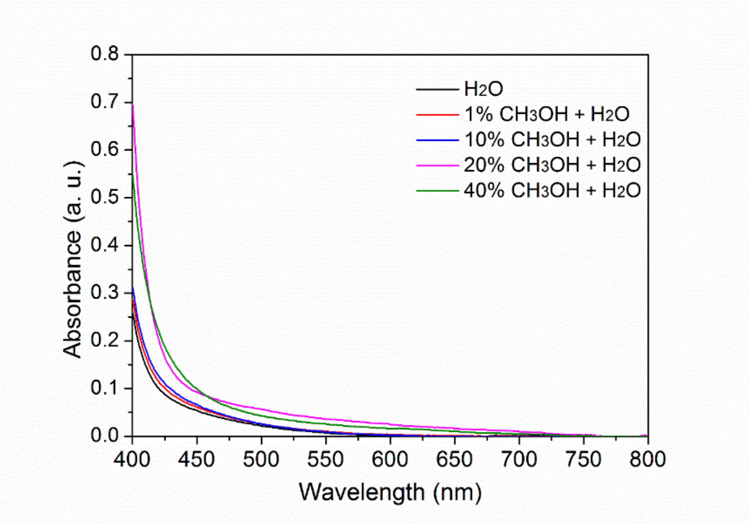

Figure S8. UV-vis absorption spectra for ammonia solutions of the same concentration (600 μg L^{-1}) containing different concentrations of methanol detected by the Nessler's reagent method.

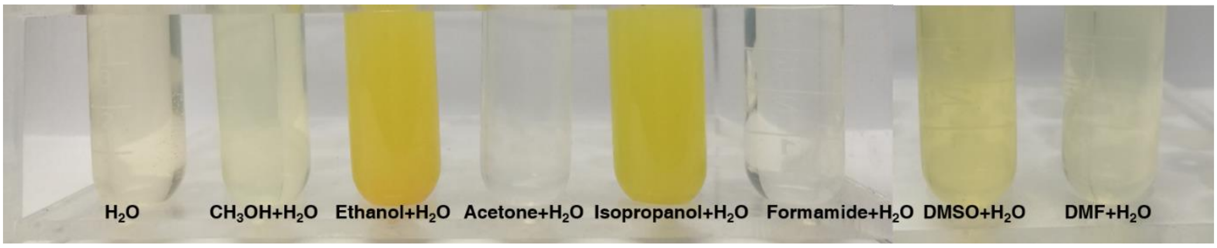

Figure S9. Photograph of various solutions using the Nessler's reagent method.

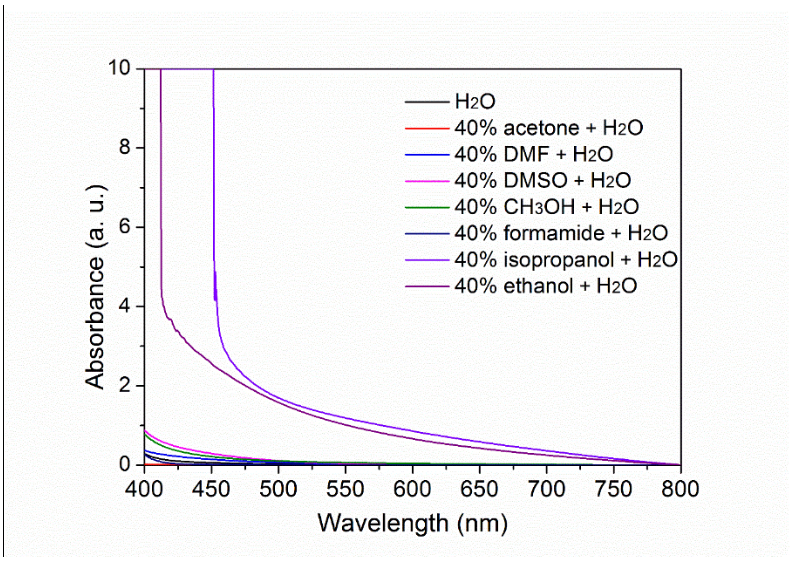

Figure S10. UV-vis absorbance spectra for ammonia solutions of the same concentration (600 μg L^{-1}) containing different organic solvents detected using the Nessler's reagent method.

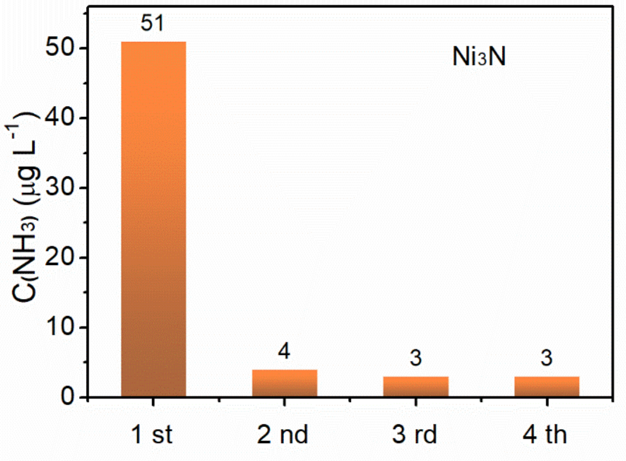

Figure S11. Ammonia concentration of aqueous solutions containing Ni_3N before and after washing with ultrapure water.

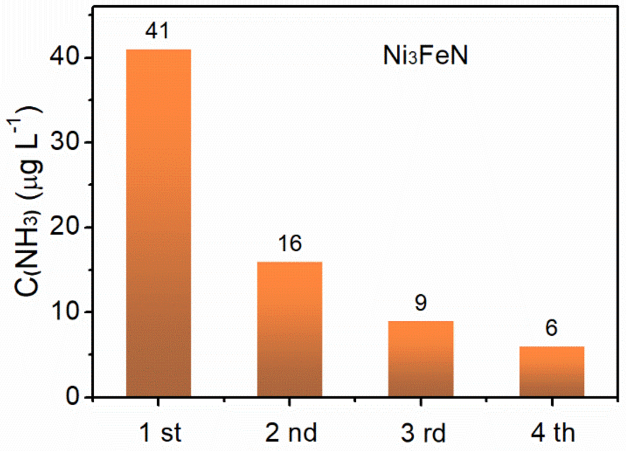

Figure S12. Ammonia concentration of aqueous solutions containing Ni_3FeN before and after washing with ultrapure water.

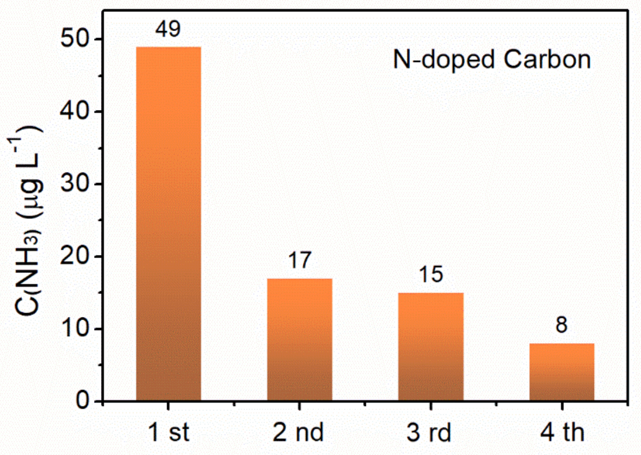

Figure S13. Ammonia concentration of aqueous solutions containing N-doped Carbon before and after washing with ultrapure water.

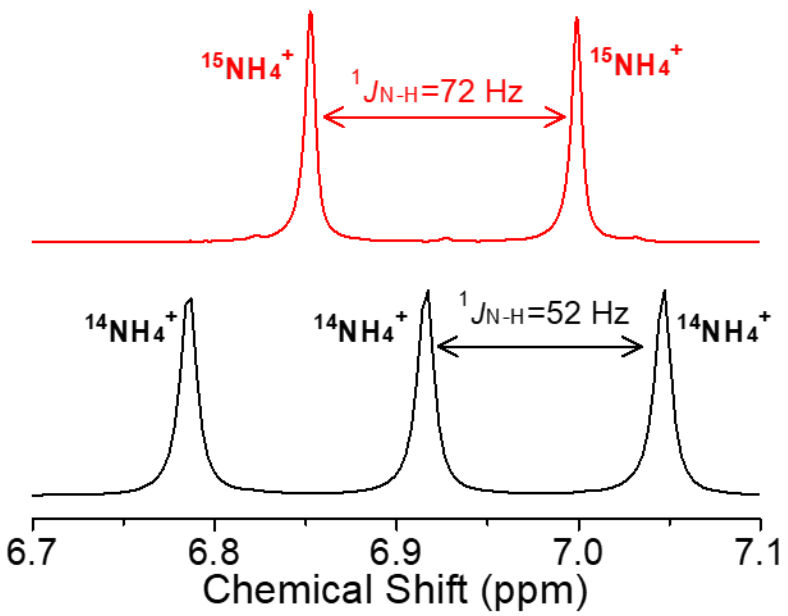

Figure S14. ^{1}H NMR analysis of 20% DMSO-d$_6$ solutions containing ($^{15}NH_4)_2SO_4$ and $^{14}NH_4Cl$ (1 mg L^{-1}).

The origin of nitrogen in photocatalytic and electrocatalytic experiments can be convincingly established using isotopically labelled $^{15}N_2$, with the $^{15}NH_4^+$ product detected with high sensitivity by ^{1}H NMR or mass-spectroscopy. Due to the presence of water, FT-IR detection of $^{15}NH_4^+$ is challenging. It is worth noting that there is low level contamination from isotope labeled gases due to the special synthetic process of $^{15}N_2$, which should be of particular concern.

Due to the typically low concentrations of $^{15}NH_4^+$ in the reaction solution and the high cost of $^{15}N_2$, the sensitivity of the testing equipment to $^{15}NH_4^+$ should first be ascertained. It is recommended that ($^{15}NH_4)_2SO_4$ and $^{14}NH_4Cl$ are obtained from a commercial supplier, such as Aladdin, as reference materials. Aquoeus solutions of ammonia containing specific concentrations of $^{15}NH_4^+$ and $^{14}NH_4^+$ can then be prepared and used to test the sensitivity of the instrument and develop NH_4^+ quantification protocols. Figure S14 shows 1H NMR spectra for 1 mg L^{-1} solutions of $^{15}NH_4^+$ and $^{14}NH_4^+$. The data show that $^{14}NH_4^+$ produces ^{14}N a triplet in the region near 6.8~7.05 ppm, whereas $^{15}NH_4^+$ appears as a doublet in the same region.

For NH_3 production via electrocatalysis, the reactor, electrode and catalyst (if used for cycling tests) require repeated cleaning with ultra-pure water or acid to remove residual ammonia before commencing a test run. Then, $^{14}N_2$ should be used to screen catalysts and optimize reaction conditions. If the amount of ammonia denerated during the reaction is sufficient to exceed the detection limit of ^{1}H NMR, then that method should be used to confirm $^{14}NH_4^+$ formation. Finally, $^{14}N_2$ should be replaced with $^{15}N_2$ and the electrocatalytic

reaction perfromed under the same reaction conditions, with the products again analyzed by ^{1}H NMR to confirm that N_2 was the origin of the NH_4^+ formed.

References

[1] Y. Zhao, Y. Zhao, G. I. Waterhouse, L. Zheng, X. Cao, F. Teng, L. Z. Wu, C. H. Tung, D. O'Hare, T. Zhang, *Adv. Mater*. **2017**, *29*, 1703828.

[2] X. Li, W. Wang, D. Jiang, S. Sun, L. Zhang, X. Sun, *Chem. Eur. J.* **2016**, *22*, 13819.

[3] Q. Wang, L. Shang, R. Shi, X. Zhang, G. I. N. Waterhouse, L.-Z. Wu, C.-H. Tung, T. Zhang, *Nano Energy* **2017**, *40*, 382.

[4] H. Yu, R. Shi, Y. Zhao, T. Bian, Y. Zhao, C. Zhou, I. N. Waterhouse Geoffrey, L.-Z. Wu, C.-H. Tung, T. Zhang, *Adv. Mater.* **2017**, *29*, 1605148.

[5] M.-M. Shi, D. Bao, B.-R. Wulan, Y.-H. Li, Y.-F. Zhang, J.-M. Yan, Q. Jiang, *Adv. Mater.* **2017**, *29*, 1606550.
